# Supplementary material for: Bypassing ubiquitination enables LAT recycling to the cell surface and enhanced signaling in T cells
Source: PLoS One. 2020 Feb 21;15(2):e0229036. doi: 10.1371/journal.pone.0229036 (PMC7034843; doi:10.1371/journal.pone.0229036)
Supplement: S1 Fig — (PDF) [file pone.0229036.s001.pdf]

Supplementary Figure 1

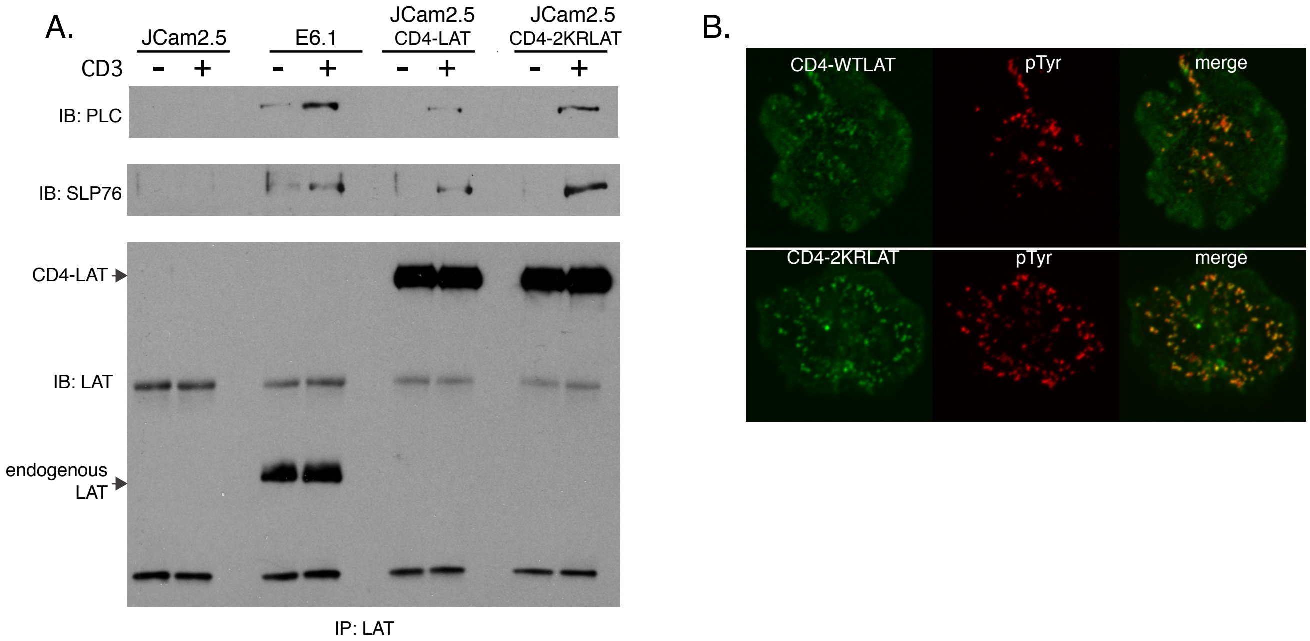

Supplementary Figure 1: Characterization of CD4-2KRLAT. A. Indicated cells were stimulated with 10µg /ml OKT3 for 2 minutes following which cell lysates were prepared. LAT was immunoprecipitated from lysates. Immunoprecipitates were blotted for LAT, PLC-γ1 or SLP-76 as indicated. Data is representative of two independent experiments. **B.** JCam2.5 cells stably expressing CD4-LAT or CD4-2KRLAT were labeled with anti-CD4 antibody conjugated to Alexa 488 for 30 minutes at 4°C. After extensive washing to remove excess antibody, cells were dropped onto anti- CD3 coated coverslips at 37°C and fixed after 2 minutes. Post-fixation, cells were permeabilized and immunostained for phosphotyrosine. Data is representative of three independent experiments.
