# Supplementary material for: Bypassing ubiquitination enables LAT recycling to the cell surface and enhanced signaling in T cells
Source: PLoS One. 2020 Feb 21;15(2):e0229036. doi: 10.1371/journal.pone.0229036 (PMC7034843; doi:10.1371/journal.pone.0229036)
Supplement: S2 Fig — (PDF) [file pone.0229036.s002.pdf]

Supplementary Figure 2

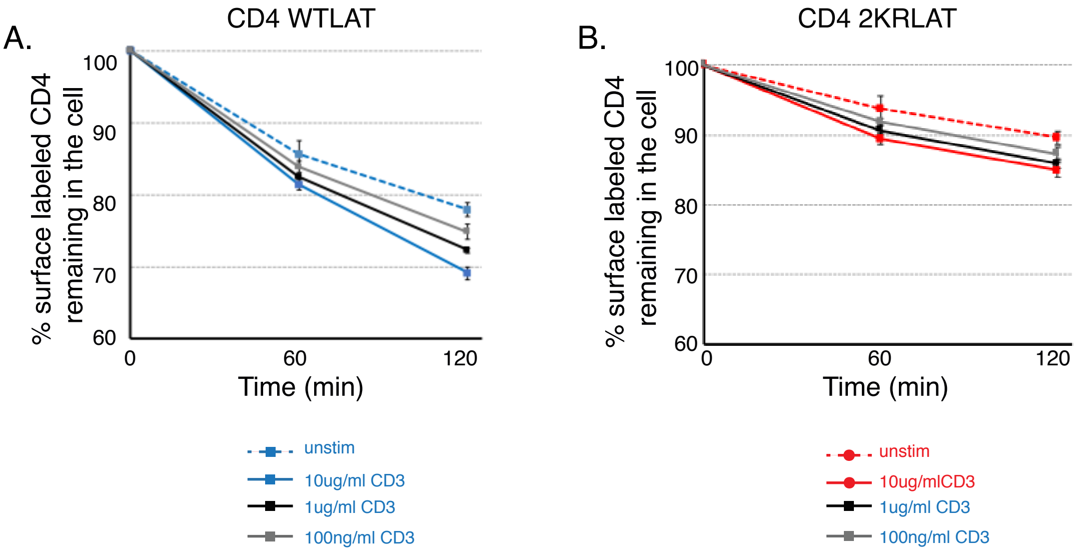

Supplementary Figure 2: Dose-dependent loss of surface LAT. JCam2.5 cells stably expressing CD4-WTLAT or CD4-2KRLAT were labeled with anti-CD4 (clone OKT4) at 4°C. Cells were left unstimulated or stimulated with indicated doses of anti-CD3 and transferred to 37°C for indicated times at which total CD4 levels were measured by flow cytometry. Data is representative of three independent experiments. % surface labeled CD4 remaining in the cell was measured as described in Materials and Methods. Bars denote means ± SEM of three independent experiments. **A.** Loss of CD4-WTLAT is increased upon anti-CD3 stimulation in a dose-dependent manner. **B.** Loss of CD4-2KRLAT is not stimulation or dose-dependent.
