# Supplementary material for: Bypassing ubiquitination enables LAT recycling to the cell surface and enhanced signaling in T cells
Source: PLoS One. 2020 Feb 21;15(2):e0229036. doi: 10.1371/journal.pone.0229036 (PMC7034843; doi:10.1371/journal.pone.0229036)
Supplement: S3 Fig — (PDF) [file pone.0229036.s003.pdf]

Supplementary Figure 3

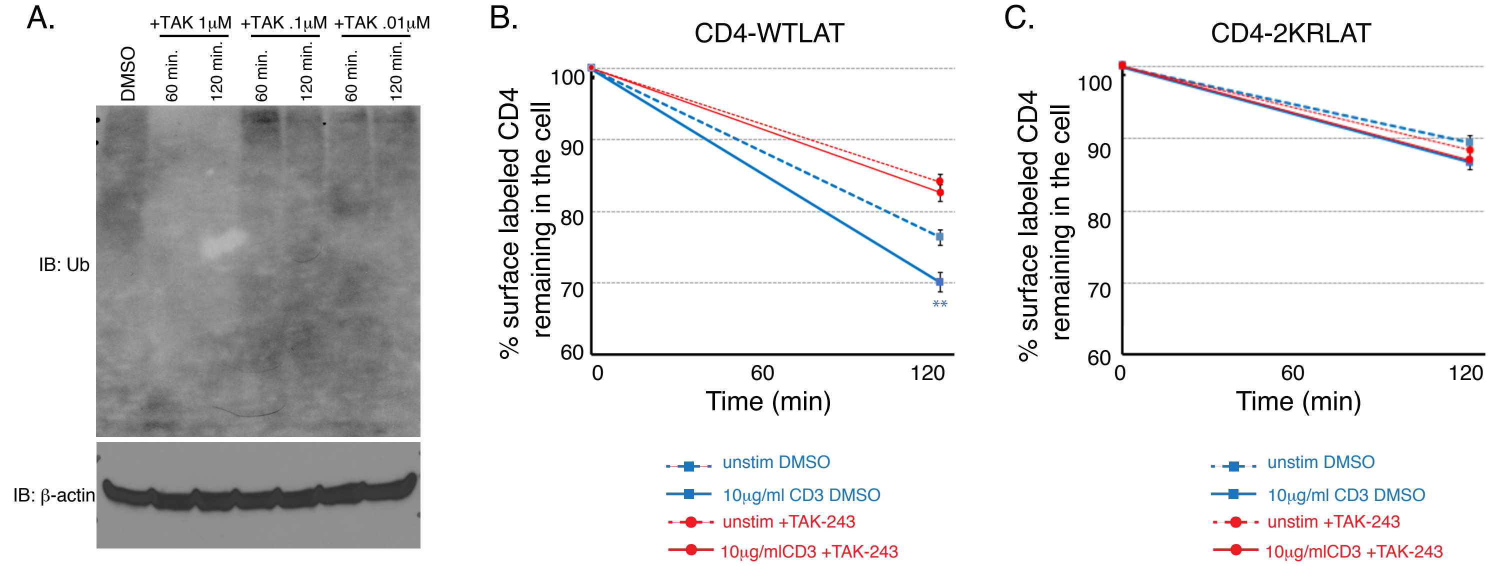

Supplementary Figure 3: E1 inhibition of cells expressing CD4-WTLAT or CD4-2KRLAT. A. JCam2.5 cells stably expressing CD4-WTLAT or CD4-2KRLAT were treated with DMSO or indicated concentrations of TAK-243 E1 inhibitor for 60 or 120 min as indicated. Whole cell lysates were immunoblotted for ubiquitin (Ub) and β-actin. **B and C.** JCam2.5 cells stably expressing CD4-WTLAT or CD4-2KRLAT were treated with 1uM TAK-243 for 60 min and labeled with anti-CD4 (clone OKT4) at 4°C. Cells were left unstimulated or stimulated with indicated doses of anti-CD3 and transferred to 37°C for 120 min. at which total CD4 levels were measured by flow cytometry. Data is representative of three independent experiments. % surface labeled CD4 remaining in the cell was measured as described in Materials and Methods. Bars denote means ± SEM of three independent experiments. Statistical significance was determine using Student's t test. ** $p < 0.0005$.
