# Supplementary figures and images for: Bypassing ubiquitination enables LAT recycling to the cell surface and enhanced signaling in T cells
Source: PLoS One. 2020 Feb 21;15(2):e0229036. doi: 10.1371/journal.pone.0229036 (PMC7034843; doi:10.1371/journal.pone.0229036)

Supplementary Figure 4

A. Blots for Supplementary Figure 1A.

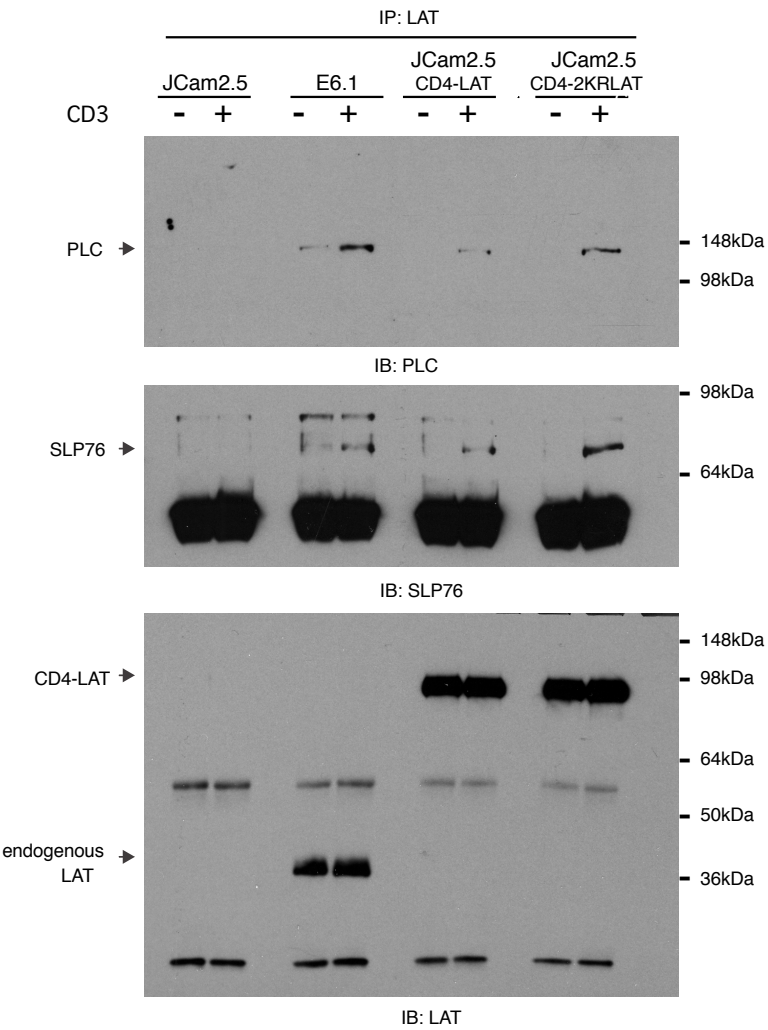

B. Blots for Supplementary Figure 3A.

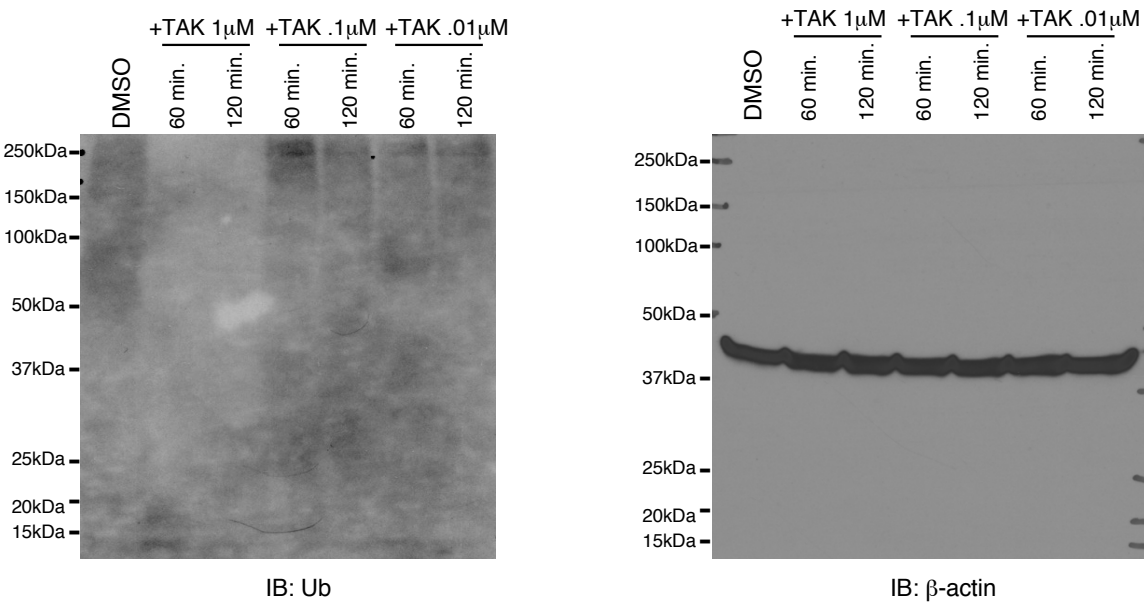

Supplement: S4 Fig — (PDF) [file pone.0229036.s004.pdf]
