# Supplementary material for: Bypassing ubiquitination enables LAT recycling to the cell surface and enhanced signaling in T cells
Source: PLoS One. 2020 Feb 21;15(2):e0229036. doi: 10.1371/journal.pone.0229036 (PMC7034843; doi:10.1371/journal.pone.0229036)
Supplement: Attachment — Submitted filename: Response to Reviewers.docx [file pone.0229036.s005.docx]

**Academic Editor Review:**

1. Our internal editors have looked over your manuscript and determined that it is within the scope of our Autophagy and Proteostasis Call for Papers. This collection of papers is headed by a team of Guest Editors: Sharon Tooze, Fulvio Regiori and Thorsten Hoope. The Collection will encompass a diverse range of research articles from early initiation of autophagy, to understand the role other proteostasis pathways play in maintaining cellular homeostasis and the cross talk between the two.  Additional information can be found on our announcement page: <https://collections.plos.org/s/autophagy-proteostasis>..
If you would like your manuscript to be considered for this collection, please let us know in your cover letter and we will ensure that your paper is treated as if you were responding to this call. If you would prefer to remove your manuscript from collection consideration, please specify this in the cover letter.

We are happy to be considered for publication in the “Autophagy and Proteostasis” Issue

2. We note that you have included the phrase “data not shown” in your manuscript. Unfortunately, this does not meet our data sharing requirements. PLOS does not permit references to inaccessible data. We require that authors provide all relevant data within the paper, Supporting Information files, or in an acceptable, public repository. Please add a citation to support this phrase or upload the data that corresponds with these findings to a stable repository (such as Figshare or Dryad) and provide and URLs, DOIs, or accession numbers that may be used to access these data. Or, if the data are not a core part of the research being presented in your study, we ask that you remove the phrase that refers to these data.

We have removed this phrase from our manuscript as the data was not required for the study.

3. PLOS ONE now requires that authors provide the original uncropped and unadjusted images underlying all blot or gel results reported in a submission’s figures or Supporting Information files. This policy and the journal’s other requirements for blot/gel reporting and figure preparation are described in detail at <https://journals.plos.org/plosone/s/figures#loc-blot-and-gel-reporting-requirements> and <https://journals.plos.org/plosone/s/figures#loc-preparing-figures-from-image-files>. When you submit your revised manuscript, please ensure that your figures adhere fully to these guidelines and provide the original underlying images for all blot or gel data reported in your submission. See the following link for instructions on providing the original image data: <https://journals.plos.org/plosone/s/figures#loc-original-images-for-blots-and-gels>

In your cover letter, please note whether your blot/gel image data are in Supporting Information or posted at a public data repository, provide the repository URL if relevant, and provide specific details as to which raw blot/gel images, if any, are not available. Email us at [plosone@plos.org](mailto:plosone@plos.org) if you have any questions.

We have included the original uncropped western blots in Supplementary Figure 4

4. Please include captions for your Supporting Information files at the end of your manuscript, and update any in-text citations to match accordingly. Please see our Supporting Information guidelines for more information: <http://journals.plos.org/plosone/s/supporting-information>.

Done

**Reviewers’ Comments**

**Reviewer #1:** The underlying molecular interactions at the initiation of the T cell response has been widely studied. One of the molecules that is implicated in the T cell response is Linker of Activation of T cells (LAT). The mechanism that underlies the turnover of LAT is the focus of the studies in this manuscript. LAT levels at the plasma membrane, its endocytosis after phosphorylation and its recycling back to the cell surface are all points in LAT turnover that can affect the T cell response. There are 2 pools of LAT; at the cell surface and in vesicular compartments. The mobilization of LAT may be regulated by its ubiquitination. In this manuscript, the authors assess the role of ubiquitination on LAT turnover.
To assess ubiquitination, a chimeric molecule was constructed that contained the extracellular domain of CD4 and the cytoplasmic domain of LAT with or without mutated lysines. Following CD4 ligation with anti CD4 antibody or cell activation with anti-CD3, they monitored the redistribution of the chimeric CD4/LAT molecule within the cell. The results of the experiments are clearly presented.
Within the context of these experiments, as an additional evaluation of the ubiquitination of LAT, experiments in the presence of cell permeable ubiquitin inhibitors that target the ubiquitin proteasome system at an early point (an E1 inhibitor) or later in the process (proteasome inhibitor) would be informative. The expectation is that the CD4WTLAT and 2KRLAT will be differentially affected. Those differences should complement the studies reported here.

We thank this Reviewer for carefully reading our manuscript and the insightful recommendation. To address the comment above, we treated Jurkat cells with TAK-243, an E1 activating enzyme inhibitor. We determined that overall cellular ubiquitination was inhibited by treatment of Jurkat cells at a dose of 1uM for 1 hr (Supp Figure 3A). We then treated Jcam2.5 cells stably expressing CD4-WTLAT and CD4-2KRLAT with 1uM TAK-243 for 1 hr and evaluated the loss of cell surface LAT. While TAK-243 treatment did not cause a change in the kinetics of loss of CD4-2KRLAT, loss of CD4-WTLAT was affected. Surface labeled CD4-WTLAT molecules were lost at a significantly lower rate in TAK-243 treated cells and TCR stimulation did not cause an increase in loss of cell surface LAT (Supp Figure 3B). Thus, in cells treated with the E1 inhibitor, WTLAT behavior resembled that of 2KRLAT. These data strengthen the conclusion from Figure 1 that degradation of cell surface LAT is ubiquitin dependent.

For targeting the ubiquitination system at a later point, we have included data from leupeptin-treated cells in the manuscript (Figure 2). We apologize that this was unclear in the first draft of the manuscript and have clarified this by including “leupeptin treatment” in the text above Figure 2A and the corresponding text in the Results section. In brief, leupeptin treatment caused accumulation of CD4-WTLAT, but not CD4-2KRLAT in lysosomes. Thus, as the reviewer predicted WT and 2KRLAT were differentially affected by leupeptin, an inhibitor that inhibits degradation of monoubiquitinated cargo at the lysosome.

**Reviewer #2:** This manuscript needs some revision as there are mistakes /inaccurate statements. Below is what I found. Authors need to proof-read it carefully.

We thank this Reviewer for carefully reading our manuscript. We have addressed the concerns listed below to the best of our ability.

Line 57: Why capitalized Cell

We have capitalized T Cell antigen Receptor to highlight the letters in the acronym TCR.

Line 58: Phosphorylation of the TCR on cytosolic tyrosine residues ...... Really ?

We have clarified this sentence by adding “Phosphorylation of the TCR *complex*…” since tyrosines in ITAMS of the zeta chain and CD3 are phosphorylated.

Line 61: Wrong reference. The first author seems to love to cite her own papers.

We have added a new reference here Courtney et al. 2018.

Line 66: leading to activation of the downstream kinases and transcription factors......what kinases down of LAT? why only kinases and transcription factors? Please rewrite this sentence.

We have rewritten this as “downstream effectors” in place of kinases and transcription factors.

Line 67: effector functions as T cell proliferation and cytokine expression. Is T cell proliferation an effector function?

We have removed “effector function” and replaced it with “outcomes”

Line 68: wrong reference

We have referenced one of our reviews here that describes LAT signaling complexes and how they mediate T cell signaling outcomes and believe that this is an appropriate reference here.

Line 81: a defect in protein turnover rate. How a turnover rate could be defective? Should be either decreased or increased!

We have replaced “defect” with “decreased” here.

Line 84: enhance T-cell potency. why we are talking potency here? potent in doing what?

We have replaced “enhance T-cell potency” with “increased T-cell signaling” here.

Line 92: move some cited references that reported essential role of surface LAT.

We have moved the cited references to the appropriate locations in the sentence.

Line 107: LAT endocytosis in these cells. What are these cells? Did you talk about any cells in this paragraph?

We have removed “these cells” from this sentence.

Line 110: Molecular switch? a fancy word. perhaps simply targets LAT for degradation.

We have removed “molecular switch” from this sentence.

Line 272: significantly effecting. should be affecting.

We have corrected this error.

Line 276-277: make it clearer for " dose dependent and TCR dose"

We have included “TCR” dose dependent to clarify this point.

Line 277: ubiquitin is a signal for the loss of cell surface LAT. Weird statement

We have rewritten this sentence to read that “the loss of cell surface LAT is ubiquitin dependent”

Line 300: labeling of the construct at the cell surface. Construct?

We have rewritten this to read “labeling of CD4-2KRLAT at the cell surface”
